# Effects of *Oenanthe javanica* on Nitrogen Removal in Free-Water Surface Constructed Wetlands under Low-Temperature Conditions

**DOI:** 10.3390/ijerph16081420

**Published:** 2019-04-19

**Authors:** Siyuan Song, Penghe Wang, Yongxia Liu, Dehua Zhao, Xin Leng, Shuqing An

**Affiliations:** 1 Institute of Wetland Ecology, School of Life Science, Nanjing University, Nanjing 210046, China; xiaoyuanlisa@163.com (S.S.); wangpenghe0610222@163.com (P.W.); Lyx1901@163.com (Y.L.); lengx@nju.edu.cn (X.L.); anshq@nju.edu.cn (S.A.); 2 Nanjing University Ecology Research Institute of Changshu (NJUecoRICH), Changshu 215500, China; 3 Shanghai Investigation, Design & Research Institute Co., Ltd. (SIDRI), Shanghai 200434, China

**Keywords:** rhizospheric microorganism, community composition, gene abundance, carbon source, C/N ratio, nitrification–denitrification

## Abstract

To investigate the role and microorganism-related mechanisms of macrophytes and assess the feasibility of *Oenanthe javanica* (Blume) DC. in promoting nitrogen removal in free-water surface constructed wetlands (FWS-CWS) under low temperatures (<10 °C), pilot-scale FWS-CWS, planted with *O. javanica*, were set up and run for batch wastewater treatment in eastern China during winter. The presence of macrophytes observably improved the removal rates of ammonia nitrogen (65%–71%) and total nitrogen (41%–48%) (*p* < 0.05), with a sharp increase in chemical oxygen demand concentrations (about 3–4 times). Compared to the unplanted systems, the planted systems not only exhibited higher richness and diversity of microorganisms, but also significantly higher abundances of bacteria, ammonia monooxygenase gene (amoA), nitrous oxide reductase gene (nosZ), dissimilatory cd1-containing nitrite reductase gene (nirS), and dissimilatory copper-containing nitrite reductase gene (nirK) in the substrate. Meanwhile, the analysis of the microbial community composition further revealed significant differences. The results indicate that enhanced abundances of microorganisms, and the key functional genes involved with nitrogen metabolism in the planted systems played critical roles in nitrogen removal from wastewater in FWS-CWS. Furthermore, abundant carbon release from the wetland macrophytes could potentially aid nitrogen removal in FWS-CWS during winter.

## 1. Introduction

Free-water surface constructed wetlands (FWS-CWS) consist of basins or channels with a suitable medium, such as soil and sand, for macrophyte rooting, and typically have water depths less than 0.4 m and hydraulic loading rates (HLR) between 0.7 and 5.0 cm·d^−1^ [1,2]. In recent years, FWS-CWS have been increasingly applied as part of an integrated wastewater treatment train and as a “stand-alone” wastewater treatment technology because of their high economy and removal efficiency [1,3]. Previous studies have indicated that FWS-CWS can achieve a removal efficiency of over 70% for total suspended solids, chemical oxygen demand (COD), biochemical oxygen demand (BOD), and pathogens, and of typically 40%–50% and 40%–90% for N and P, respectively [2,4]. The purification processes in FWS-CWS mainly occur through complex interactions between macrophytes and the associated microorganisms in the water phase [2,4]. The major pathway for nitrogen removal in the FWS-CWS is nitrification–denitrification. During nitrification, the nitrifying bacteria oxidize ammonia under aerobic conditions, while during denitrification, nitrate is converted to free nitrogen or nitrous oxide by denitrifying bacteria in the anoxic zones [5,6,7].

It is generally assumed that wetland macrophytes are closely related to the abundance, activity, and diversity of the rhizospheric microorganisms in FWS-CWS [4,5,8]. The macrophytes provide root surface for the growth of microorganisms in the rhizosphere [9,10]. They also provide root exudates or plant litter as a source of carbon compounds for heterotrophic bacteria [5,8,11]. In addition, aquatic macrophytes can deliver oxygen to the rhizosphere by radial oxygen loss (ROL) [12], thus affecting the redox status of wetland sediments and the aerobic microorganisms [2,5,13,14]. However, some researchers have concluded that macrophytes rarely affect the microbial community composition, abundance, and specific microbial functional genes [15,16,17,18]. Furthermore, some studies have indicated a limited or even negligible influence of wetland macrophytes on the nutrient removal from wastewater in constructed wetlands (CWs) under certain conditions [19,20]. Previous studies have also indicated that the exact effects of macrophytes in CWs are complex and remain disputed. Therefore, further research on the detailed mechanisms, especially the microbiological mechanisms, of nutrient removal from wastewater in FWS-CWS by macrophytes is necessary to help elucidate the exact role of macrophytes in CWs. Further, the temperature is an important factor influencing the wastewater treatment in FWS-CWS. Nutrient removal from wastewater remains a challenge in North China where the average water temperature during winter is lower than 10 °C, resulting in declined biotic activity [21,22,23]. Hence, the selection of suitable macrophyte species to mitigate the decrease in system purification capacity during winter merits attention.

The most common species used in FWS-CWS include those from the genera *Typha*, *Scirpus*, *Phragmites*, *Juncus*, and *Eleocharis* [3,24,25]. *Oenanthe javanica* (Blume) DC., a native aquatic macrophyte of China, has been proposed as an ideal candidate for nitrogen removal in CWs during the low-temperature season because of its advantages, such as fast growth in wastewater, tolerance to freezing temperatures, and capacity for repeated harvest [26]. However, studies on the potential of *O. javanica* for wastewater purification in FWS-CWS are still limited. In particular, the underlying microorganism-related mechanisms influencing nitrogen removal by *O. javanica* roots in FWS-CWS during low-temperature seasons are poorly investigated.

In the present study, FWS-CWS planted with *Oenanthe javanica* (Bl.) DC. as well as other control systems, were set up and fed with effluents from a secondary wastewater treatment plant (WWTP) during the low-temperature season. The nutrient removal performances were measured and compared among the different treatment systems. Plant growth dynamics, root physiological characteristics, abundances of the key functional genes involved in the nitrogen removal process as well as the microbial abundances, diversity, and community composition in the substrate were investigated to establish the exact role and detailed mechanism of nutrient removal by macrophytes in FWS-CWS. This is the first study that systematically explains the effects of *O. javanica* on the nitrogen removal in WS-CWS under low-temperature conditions.

## 2. Materials and Methods

### 2.1. Experimental Design

**Figure A1:**
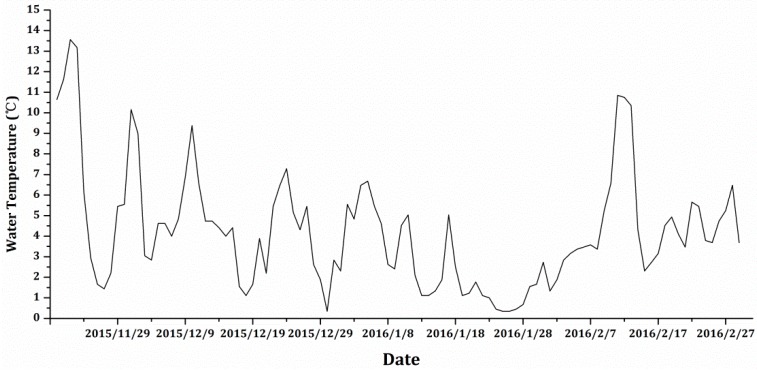
Daily water temperature of the systems during the pre-processing and experimental periods.

To investigate the precise role of macrophytes in facilitating nitrogen removal from wastewater in FWS-CWS during winter, different wastewater purification systems were built via several fiber-reinforced plastic incubators (2.1 m in length, 1.3 m in width, and 0.65 m in height) in Huai’an, Jiangsu Province, Eastern China (33.3° N, 119.0° E) on 20 November 2015. These systems were: (1) FWS-CWS planted with *O. javanica* and substrate sand (Tcw); (2) control systems without macrophytes but with substrate sand (Tcs) [27]; (3) control systems planted with *O. javanica* but without the substrate sands (Tcp); (4) control systems with blank incubators filled with wastewater only (Tck). Each system included four replicates. Cleansed sand (1–2 mm in diameter; 15 cm in thickness) was used as the substrate in Tcw and Tcs. The water level of each system was 35 cm. Several concrete bricks wrapped in polyethylene bags were used in Tcp to maintain the same liquid height as the other two groups. Planted seedlings of *O. javanica* with similar size (65 ± 2.5 cm in length) were selected from a local nursery and cleaned to remove the rhizospheric soil. The initial density was 26 plants per m^2^ (72 plants per incubator). The secondary wastewater was obtained from a neighboring WWTP and their primary characteristics are listed in Table 1. The experiments were performed as a batch model (i.e., wastewater was filled to a liquid height of 0.3 m at the beginning of each batch and then drained before the next batch). Each batch lasted 10 days (hydraulic retention time = 10 days). At the end of each batch, the water level of each system was measured to calculate changes in the water volume. There was a total of eight batches (80 days) from 10 December 2015 to 29 February 2016 in this study. A Temperature/Light Data Logger (HOBO UA-002-08; Onset, Cape Cod, MA, USA) was used to record the water temperature. The changes in water temperature during the experiment are shown in Figure A1.

### 2.2. Water and Plant Sampling and Analysis

The water in each system was sampled for water quality at the end of the 2nd, 4th, 6th, and 8th batches. The water temperature, pH, and dissolved oxygen (DO) were measured by a Temperature/Light Data Logger (HOBO UA-002-08; Onset, Cape Cod, MA, USA), a portable Multi-parameter Water Quality Meter (U52; Horiba Ltd., Kyoto, Japan) and a DO electrode (HQ40D-53LED; Hach Company, Loveland, CO, USA), respectively. NH_4_^+^–N, NO_2_^−^–N, NO_3_^−^–N, total nitrogen (TN), and COD were determined through water quality analyzing systems (DRB200 and DR2800; Hach Company, Loveland, CO, USA) according to standard analytical procedures [28]. The water sampling was conducted according to guidelines on sampling from lakes, natural and man-made (ISO/FDIS 5667-4:2016).

Biomass and nitrogen content of plant samples from the beginning and end of the experiment were determined according to [29]. Briefly, plant samples were separated into roots, stems, and leaves, dried at 65 °C to a constant weight, grounded into powder, and then measured by an elemental analyzer (CHN-O-Rapid; W. C. Heraeus GmbH., Hanau, Germany) [30]. Root vitality was quantified with the triphenyl tetrazolium chloride (TTC) method [29]. The rate of root ROL was measured through the titanium (III) citrate buffer method [31,32].

### 2.3. Microorganism Sampling and Analysis

#### 2.3.1. Preparation of Microbial Samples

The microbial samples from the rhizoplane of wetland macrophytes and the substrates were obtained on 10 January 2016. For the rhizoplane samples, 5 g of root was obtained from five plants in each system and placed into clean phosphate buffer in a Falcon tube. After ultrasonic processing at 90 W for 30 min, the isolated biofilm from the rhizoplane was collected by vacuum filtration with 0.22 μm membranes [33]. For substrate samples, 100 g of sand was obtained using a cylindrical sampler (diameter, 0.5 cm) from five sampling points in each system and added to a sterile glass bottle, then mixed well, and vigorously shaken at 200 rpm for 3 h to isolate the biofilm. After the centrifugation process of samples at 5000× g for 12 min, the precipitate was collected for analysis [34]. Biofilm isolated from the rhizoplane and the substrate were used for subsequent DNA extraction, qPCR, 16S rRNA gene PCR amplification, and Illumina MiSeq sequencing.

#### 2.3.2. Extraction of Total Genomic DNA

The total genomic DNA from microbial samples was first extracted and purified using QIAamp Fast DNA Stool Mini Kit (QIAGEN, Chatsworth, CA, USA) and the yield was evaluated with SpectraMax 190 (Molecular Devices, Sunnyvale, CA, USA). Subsequently, the integrity was detected with 1% agarose gel electrophoresis and stored at −20 °C until further use.

#### 2.3.3. Real-Time Quantitative PCR Analysis

Quantitative PCR analysis of seven target functional gene fragments, (i.e., bacteria (bacterial 16S rRNA gene), archaea (archaeal 16S rRNA gene), anaerobic ammonia oxidation (anammox), bacteria (ANO 16S rRNA gene), ammonia monooxygenase gene (*amoA*), nitrous oxide reductase gene (*nosZ*), dissimilatory cd1-containing nitrite reductase gene (*nirS*), and dissimilatory copper-containing nitrite reductase gene (*nirK*) was conducted using the Illumina-Eco real-time PCR system (Illumina, San Diego, CA, USA). The primers were synthesized by Genergy Biotechnology Limited Corporation (Shanghai, China) and the details are listed in Table A1. Further information regarding the qPCR analysis is shown in Table A2.

#### 2.3.4. 16.S rRNA Gene Illumina MiSeq Sequencing

PCR amplification of the 16S rRNA gene was performed with the universal primer set at 341F (5′-CCTAYGGGRBGCASCAG-3′) and 785R (5′-GACTACHVGGGTATCTAATCC-3′). Raw fastq files were demultiplexed, quality-filtered, and merged by FASTX-Toolkit (version 0.0.14) and Mothur program (version 1.34.0,) [35]. All reads were quality filtered using an average quality value of 20 (Q20) during demultiplexing. Short reads (length <40 bp) and chimeras were excluded. Reads were clustered according to the degree of similarity by using the Uclust program (version 1.2.22q, Edgar 2010). Sequences with ≥97% similarity were assigned to the same genus. Taxonomic information was annotated by the Ribosomal Database Project (RDP) classifier (version 2.2) [36], and the alpha diversity was analyzed by Mothur. Chao1 and Simpson index were used to estimate the species richness [37] and species diversity [38,39], respectively. More information of the 16S rRNA gene Illumina MiSeq sequencing is listed in Table A2.

#### 2.3.5. Sequence Storage Information

The Illumina sequencing raw data have been deposited in the National Center for Biotechnology Information (NCBI) Sequence Read Archive database (Study Accession: SRP105263; Sample Accessions: SRS2149921, SRS2149998, SRS2149999, SRS2150553).

### 2.4. Statistical Analysis

Statistical Package for Social Sciences (SPSS) 17.0 (SPSS Inc., Armonk, NY, USA) was used for statistical analysis. The data were analyzed using a one-way analysis of variance to compare the performance of each mesocosm, and statistically significant differences (*p* > 0.05) between the mean values of the treatments were determined using Duncan’s test. Nonparametric tests were used for non-normal distribution data.

## 3. Results

### 3.1. Nutrient Removal Performance

**Figure 1:**
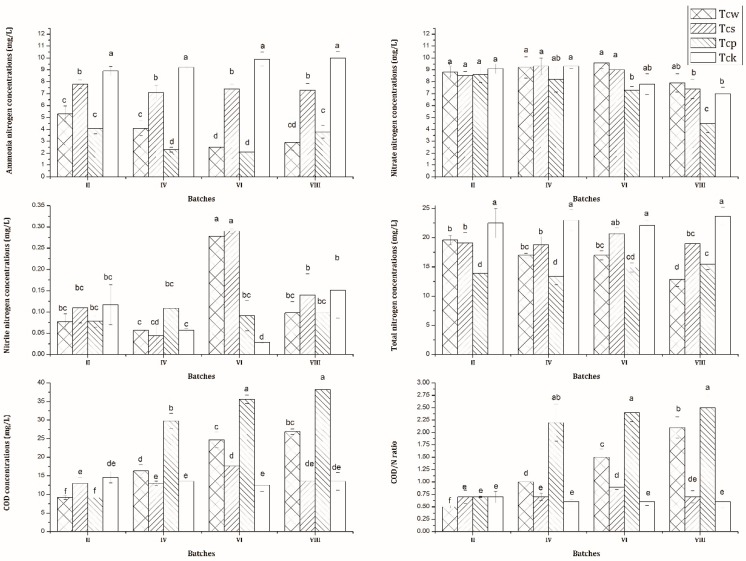
Concentrations (mg·L^−1^) of NH_4_^+^–N, NO_3_^−^–N, NO_2_^−^–N, TN, and COD as well as the COD/N ratio (*n* = 4) Different letters indicate significant differences (*p* < 0.05) among the different systems. Tcw: free-water surface constructed wetlands (FWS-CWS) planted with *O. javanica* in substrate; Tcs: control systems without plants; Tcp: control systems without sands; Tck: control systems with blank incubators filled with wastewater only. II, IV, VI, and VIII represent respectively the 2nd, 4th, 6th, and 8th batch of different systems (Tcw, Tcs, Tcp, and Tck).

The performance of nutrient removal varied greatly across the different systems (Figure 1). Tcw and Tcp, followed by Tcs (28.62%), achieved the highest average removal rate of NH_4_^+^–N (64.58%–70.68%). However, no significant differences in the average removal rates of NO_3_^−^–N and NO_2_^−^–N were observed among the three systems in the four detected batches. The best removal efficiency with regard to TN was observed in Tcp (40.57%–46.41%) in the 2nd, 4th, and 6th batches and in Tcw (48.37%) in the 8th batch. Additionally, over time, an increase in the effluent COD concentrations was observed in Tcw and Tcp. It increased from 9.2 mg·L^−1^ to 26.9 mg·L^−1^ and from 9.1 mg·L^−1^ to 38.2 mg·L^−1^ in Tcw and Tcp, respectively. Accordingly, the ratio of COD/N in the effluent sharply increased from 0.5 to 2.1 and from 0.7 to 2.5 in Tcw and Tcp, respectively.

### 3.2. Plant Growth Dynamics and Physiological Root Characteristics

Table 2 shows the plant lengths, biomass, nitrogen content, and root activity as well as the ROL rate. The data indicate slight plant growth during the operation; however, no obvious difference was observed among these indicators between Tcw and Tcp. First, the plant shoot lengthened by 5.0–6.0 cm while the plant root lengthened by 7.6–8.0 cm at the end of the 8th batch in Tcw and Tcp. Meanwhile, the plant shoot biomass was enhanced by 7.3–9.5 g·m^−2^ while the plant root biomass was enhanced by 11.8–13.0 g·m^−2^ during the operation period. Accordingly, the nitrogen content also increased by 0.110–0.143 g·m^−2^ and 0.108–0.117 g·m^−2^ in the shoot and root, respectively. Unlike the plant growth dynamics indicators, the physiological characteristics of plant root presented a mild fluctuation in Tcw and Tcp during the operation period. The plant root vitality showed a slight decline at the end of 2nd, 4th, and 6th batches in comparison to the beginning, but increased to 58.7–63.6 μg TTC·g^−1^ root·h^−1^ at the end of the 8th batch. Further, the ROL rates increased by 0.32–0.52 μmol O_2_·g^−1^ root·h^−1^ in Tcw and Tcp at the end of 8th batch, though they presented a slight decline at the end of the 2nd and 4th batches when compared to the beginning.

### 3.3. Microbial Population and Composition

**Figure 2:**
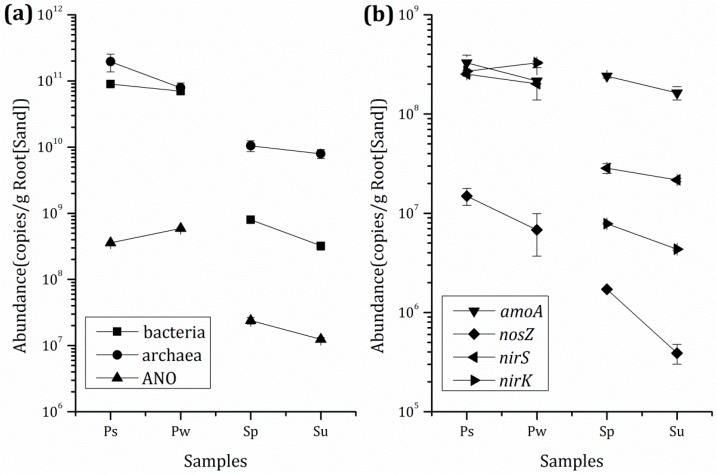
Absolute abundances of microbial communities and functional genes: (**a**) bacterial 16S rRNA, archaeal 16S rRNA, and 16S rRNA related to anammox bacteria; (**b**) *amoA*, *nosZ*, *nirS*, and *nirK* (*n* = 3). Ps: sample from rhizoplane in Tcw; Pw: sample from rhizoplane in Tcp; Sp: sample from sand in Tcw; Su: sample from sand in Tcs. The microbial samples from the plants rhizoplane and the substrate were obtained in the end of the 6th batch.

The absolute abundances of bacteria, archaea, and anammox as well as the four functional genes, (i.e., *amoA*, *nosZ*, *nirS*, and *nirK*) in the samples from plant roots or sand in the three systems are shown in Figure 2. Except for anammox and *nirK*, the abundances of bacteria, archaea, *amoA*, *nosZ*, and *nirS* in samples from rhizoplane in Tcw (Ps) were higher when compared to samples from rhizoplane in Tcp (Pw). The copy numbers in Ps were recorded as 8.947 × 10^10^ copies·g^−1^ root (bacteria), 1.954 × 10^11^ copies·g^−1^ root (archaea), 7.335 × 10^7^ copies·g^−1^ root (*amoA*), 1.491 × 10^7^ copies·g^−1^ root (*nosZ*), and 2.517 × 10^8^ copies·g^−1^ root (*nirS*), while the numbers were 7.017 × 10^10^ copies·g^−1^ root (bacteria), 7.871 × 10^10^ copies·g^−1^ root (archaea), 4.828 × 10^7^ copies·g^−1^ root (*amoA*), 6.812 × 10^6^ copies·g^−1^ root (*nosZ*), and 2.017 × 10^8^ copies·g^−1^ root (*nirS*) in Pw. On the other hand, significantly high abundances of all seven target functional gene fragments were observed in samples from sand in Tcw (Sp) when compared to samples from sand in Tcs (Su). The copy numbers of bacterial 16S rRNA, archaeal 16S rRNA, anammox bacterial 16S rRNA, *amoA*, *nosZ*, *nirS*, and *nirK* in Sp were recorded as 7.987 × 10^8^ copies·g^−1^ sand, 1.049 × 10^10^ copies·g^−1^ sand, 2.392 × 10^7^ copies·g^−1^ sand, 5. 413 × 10^7^ copies·g^−1^ sand, 1.716 × 10^6^ copies·g^−1^ sand, 2.842 × 10^7^ copies·g^−1^ sand, and 7.865 × 10^6^ copies·g^−1^ sand, respectively, while they were 3.220 × 10^8^ copies·g^−1^ sand, 7.930 × 10^9^ copies·g^−1^ sand, 1.241 × 10^7^ copies·g^−1^ sand, 3.658 × 10^7^ copies·g^−1^ sand, 3.897 × 10^5^ copies·g^−1^ sand, 2.170 × 10^7^ copies·g^−1^ sand, and 4.343 × 10^6^ copies·g^−1^ sand, respectively, in Su.

Alpha diversity analysis based on the 16S rRNA gene MiSeq sequencing shows the community composition characteristics of microorganisms from plant rhizosphere and sand in the three systems (Table A3). The community richness was analyzed by calculating the Chao1 estimator at 5% dissimilarity, while community diversity was estimated by Shannon index at 5% dissimilarity. Community evenness was indicated via Shannon-even index at 5% dissimilarity. The results showed a significant improvement in richness, diversity, and evenness of microbial communities in the plant roots of Pw in contrast to those of Ps. Meanwhile, an obvious superiority of microbial community diversity and evenness was observed for Sp in comparison to Su.

**Figure 3:**
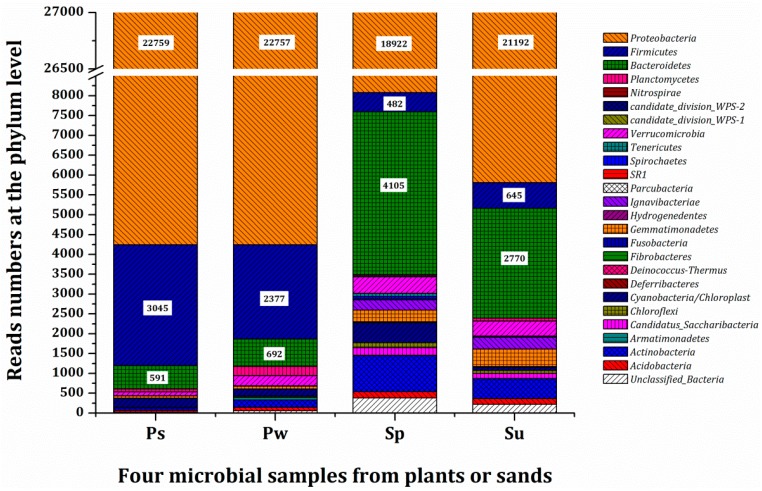
Microbial communities in the samples from plants or sands at the phylum level. Some phyla (read numbers <10) are grouped into “others”. Ps: sample from rhizoplane in Tcw; Pw: sample from rhizoplane in Tcp; Sp: sample from sand in Tcw; Su: sample from sand in Tcs. The microbial samples from the plants rhizoplane and the substrate were obtained at the end of the 6th batch.

A pairwise comparison of the bacterial community composition at the phylum level is shown in Figure 3. A total of 25 distinguishable phyla were detected, of which 0.06%, 0.21%, 1.41%, and 0.80% were unclassified reads in Ps, Pw, Sp, and Su, respectively. In Ps and Pw, the dominant phylum Proteobacteria accounted for 84.29% of the total reads, followed by Firmicutes with relative abundances of 11.28% (Ps) and 8.80% (Pw), followed by Bacteroidetes, of which, the relative abundances were recorded as 2.19% (Ps) and 2.56% (Pw). A different phylum abundance order was found in bacterial samples from Sp and Su. Though the dominant phylum was also Proteobacteria (70.08% for Sp, 78.49% for Su), the second abundant phylum was Bacteroidetes (15.20% for Sp, 10.26% for Su), followed by Actinobacteria (3.36%) in Sp and Firmicutes (2.93%) in Su.

**Figure 4:**
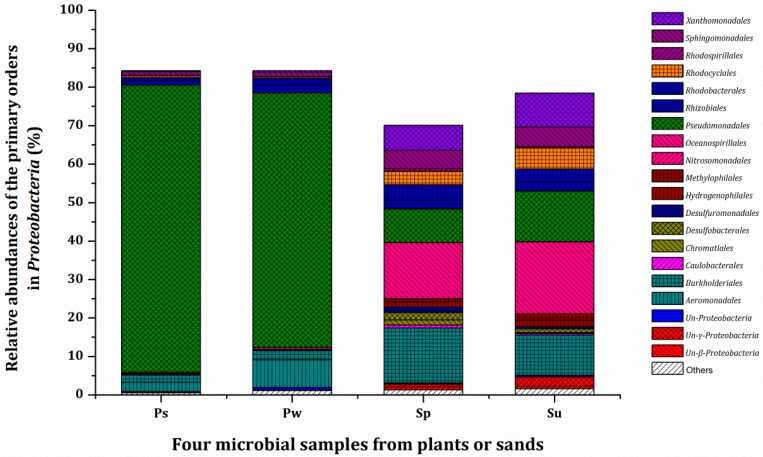
Relative abundances of the primary orders in Proteobacteria in the samples from plants or sands. Orders whose relative abundance is less than 0.5% (except for Nitrosomonadales) are grouped into “others”. Ps: sample from rhizoplane in Tcw; Pw: sample from rhizoplane in Tcp; Sp: sample from sand in Tcw; Su: sample from sand in Tcs. The microbial samples from the plants rhizoplane and the substrate were obtained at the end of the 6th batch.

Furthermore, the predominant phylum Proteobacteria was extensively analyzed by order (Figure 4). The samples from Ps and Pw shared a similar composition of order, and differences were observed only in the relative abundances of the primary orders. These were recorded as Pseudomonadales (74.70%), Aeromonadales (2.44%), Burkholderiales (1.79%), and Rhizobiales (1.12%) in Ps, and Pseudomonadales (66.15%), Aeromonadales (7.23%), Burkholderiales (2.37%), and Rhizobiales (2.09%) in Pw. The microbial samples presented larger differences between Sp and Su with regard to order composition. The predominant orders in Sp included Oceanospirillales (14.49%), Burkholderiales (14.33%), Pseudomonadales (8.80%), Xanthomonadales (6.49%), Sphingomonadales (4.76%), Rhodobacterales (4.02%), and Rhodocyclales (3.42%), while those in Su were Oceanospirillales (18.69%), Pseudomonadales (13.19%), Burkholderiales (10.42%), Xanthomonadales (8.88%), Rhodocyclales (5.41%), Sphingomonadales (5.09%), and Rhodobacterales (3.40%).

The primary genera (relative abundance >1.00%) in the four systems with the addition of two nitrifying bacteria (*unclassified-Nitrosomonadaceae* and *Nitrospira*), comprised a total of 48 genera (Table A4). According to the list of genera provided by Heylen et al. [40] and Philippot et al. [41], which included at least one denitrifying strain, nearly half of these were closely related to denitrification. Comparison of the community composition of these denitrifying bacteria showed a significant difference among the two groups of wastewater treatment systems. In Ps, *Pseudomonas*, with a relative abundance of 74.33%, was the dominant genus, followed by *Exiguobacterium* (8.89%), *Aeromonas* (2.44%), *Bacillus* (1.72%), and *Rhizobium* (0.60%). In Pw, *Pseudomonas* accounted for 65.76%, followed by *Aeromonas* (7.23%), *Exiguobacterium* (5.14%), *Paenibacillus* (2.93%), *Rhizobium* (1.01%), *Acidovorax* (0.94%), *Flavobacterium* (0.67%), and *unclassified-Rhodobacteraceae* (0.61%). The order for Sp was *Halomonas* (14.49%), *Rhodoferax* (7.73%), *Pseudomonas* (5.19%), *Arenimonas* (5.00%), *unclassified-Bacteroidetes* (4.18%), *Flavobacterium* (2.79%), *Perlucidibaca* (2.46%), *unclassified-Rhodobacteraceae* (2.20%), *Simplicispira* (1.93%), *Hydrogenophaga* (1.82%), and *Thiobacillus* (1.35%). The order for Su was *Halomonas* (18.68%), *Pseudomonas* (9.64%), *Arenimonas* (5.01%), *Rhodoferax* (3.19%), *unclassified-Xanthomonadaceae* (3.16%), *Perlucidibaca* (2.93%), *Hydrogenophaga* (2.79%), *unclassified-Bacteroidetes* (1.88%), *unclassified-Rhodobacteraceae* (1.84%), and *Thiobacillus* (1.71%). In addition, more abundant nitrifying bacteria were observed in Sp in comparison to Su. The relative abundance of *unclassified-Nitrosomonadaceae* was recorded as 0.0889% and 0.0333% in Sp and Su, respectively.

## 4. Discussion

Although the process of nitrogen transformation and removal in CWs is complex and attributed to various mechanisms including ammonification, nitrification–denitrification, anammox, vegetation uptake, biomass assimilation, dissimilatory nitrate reduction, substrate adsorption, and ammonia volatilization [10,42,43], it has been widely considered that the removal of nitrogen is primarily due to microbial metabolic pathways [44,45]. Therefore, in the current research, only the nitrogen content was measured in the wastewater as well as the macrophytes. As previously reported, the present results indicate that the vegetation uptake pathway in planted systems was limited. It was attributed to the weak growth of *O. javanica* at a low average temperature below 5 °C.

Due to the presence of diverse forms of nitrogen in wastewater, nitrogen removal in CWs often involves a series of microbial communities with a variety of metabolic functions. In general, the removal of nitrogen from wastewater is closely related to the microbial abundance and community composition which directly decides the functional characteristics of microorganisms in the CWs [46,47]. In this study, the presence of macrophytes significantly improved microbial abundances, as well as had slight lifting effects on the richness, diversity, and evenness of microbial composition, which, in turn, led to an enhanced performance of nitrogen removal. This is consistent with previous studies that reported enhanced density, activity, and diversity of microorganisms in the plant rhizosphere [48,49,50,51]. Quantitative analysis showed dramatically higher abundances of bacteria and archaea in the plant rhizoplane compared to those in the substrate of both planted and unplanted systems which suggests that it could be potentially attributed to the enlargement and complexity in attachment surface for microbial growth [10].

Nitrification includes two steps [10]. In the first step, ammonia oxidation oxidizes ammonium to nitrite, which is generally believed to be the rate-limiting step of nitrification and is usually marked by the *amoA* gene [44]. The second step, nitrite oxidation, converts nitrite into nitrate, which involves nitrite-oxidizing bacteria. The organisms that participate in ammonia oxidation mainly belong to two groups. One is ammonia-oxidizing bacteria (AOB), which have long been considered critical in nitrification, and the other is ammonia oxidizing archaea (AOA), which play an active role in nitrification by molecular biological methods. In the current study, archaeal abundances were significantly high when compared to the bacterial abundances in the planted systems. This result was consistent with several previous reports that found that AOA was the preponderant ammonia-oxidizing microorganism in the plant rhizosphere and had significantly active involvement in nitrification in the rhizosphere due to their better adaptability to the rhizosphere microenvironment in comparison to AOB [52,53,54]). Moreover, the abundances of archaea and *amoA* gene in the planted systems showed significant increases than those in the unplanted ones. The analysis of bacterial composition at different taxonomic levels also revealed a higher relative abundance of *Nitrosomonadaceae*, identified as a representative family of AOB, in planted systems compared to the unplanted ones. The results indicate that the presence of macrophytes facilitated nitrogen removal from wastewater via stimulating the abundance of ammonia-oxidizing microorganisms in the FWS-CWS. The detailed mechanism may involve the release of oxygen by roots of macrophytes which changed the partial oxidation-reduction conditions in the rhizosphere. According to the studies applying stoichiometry method, the lowest critical concentration of DO for ammonium oxidation is 1.0 mg·L^−1^ and the complete nitrification of 1.0 g ammonia nitrogen needs 4.6 g of oxygen [5]. Although there is a thin aerobic layer at the water surface due to passive diffusion from air to water, the FWS-CWS is largely an anoxic system because DO decreases with water depth and organic sedimentation at the surface of the substrate consumes a mass of oxygen [5]. Therefore, together with the low temperature, the removal of ammonia nitrogen and TN in the unplanted systems were found to be limited in the current research. In contrast, the removal of ammonia nitrogen in planted systems presented evident superiority in contrast to unplanted ones because wetland macrophytes can efficiently transfer oxygen from air to the CW system via ROL [4]. However, the advantage of TN removal was not recorded in the planted systems during the experiment. This result may be related to the limited nitrate-nitrogen removal which could be attributed to another group of the microbial community named denitrifies.

Denitrifying microorganisms exist across a wide range of microbial groups, which involve *Actinomycetes*, *Aquifaceae*, *Bacteroides*, Firmicutes, Proteobacteria, and even archaea as well as fungus [41]. A total of more than 60 genera have been identified as denitrifying bacteria and most of them belong to *Alphaproteobacteria*, *Betaproteobacteria*, and *Gammaproteobacteria* [41]. In this study, denitrifying bacteria accounted for half of the primary genera listed, which indicated the diversity of denitrifier in the FWS-CWS. *Halomonas*, *Rhodoferax*, *Pseudomonas*, and *Arenimonas* constituted the four primary genera of denitrifying bacteria in both the planted and unplanted systems. Hence, it is likely that the difference in community composition of denitrifying bacteria was not the material cause of the difference in nitrogen removal efficiency between the planted and unplanted systems. In contrast, significant differences between the planted and unplanted systems were obviously revealed by the quantitative analysis of the critical functional genes, including *nosZ*, *nirS*, and *nirK*.

Denitrification, which converts nitrate to nitrogen gas, consists of four steps and commonly occurs under anaerobic conditions [6,55]. The first step which involves converting nitrate into nitrite may occur under aerobic conditions catalyzed by Nap or anaerobic conditions catalyzed by Nar. The second step which converts nitrite into nitric oxide is catalyzed by two key enzymes: dissimilatory copper-containing nitrite reductase encoded by the *nirK* gene and dissimilatory cd1-containing nitrite reductase encoded by the *nirS* gene. The third step is the conversion from nitric oxide to nitrous oxide. The last step is catalyzed by the product of the *nosZ* gene which converts nitrous oxide into nitrogen gas. Additionally, the *nosZ* gene is often regarded as the marker of complete denitrification while the *nirS* and *nirK* genes usually act as the markers for the second denitrification step [6,55]. In the current research, the absolute abundances of the three genes, especially the *nosZ* gene, presented significant increases in the planted systems in contrast to the unplanted ones. This result indicates the remarkable facilitation of the quantity of denitrifying bacteria by wetland macrophytes which could be ascribed to the increased supply of biodegradable carbon. It is widely believed that the denitrification in CWs depends on organic carbon levels. Ye and Li [56] concluded that the entire denitrification of 1.0 g nitrate nitrogen into nitrogen gas needed 2.86 g BOD. Meanwhile, a variety of studies have shown that macrophytes can effectively enhance the carbon content, thus, aiding nitrogen removal in the CWs [7,25,57]. On the one hand, plant root exudates can provide available organic compounds to heterotrophic bacteria [8], on the other, plant litter can release various dissolved organic matters including sugars, amino acids, and volatile fatty acids [48,58]. In this study, the enhanced COD content and COD/N ratios in wastewater accompanied by the increased removal of TN from the wastewater were recorded in the planted systems. Despite the optimal COD/N ratio of 5:1 for nearly complete removal of nitrogen in the FWS-CWS [59], the elevated COD/N ratio, which reached up to 2:1 in several later batches in the planted systems, may effectively promote the accumulation of denitrifying microorganisms and stimulate denitrification, and subsequently, comprehensively improve the removal efficiency of TN from wastewater.

Besides the nitrification–denitrification process, anammox has been widely indicated as a new ammonium oxidation process which occurs under anaerobic conditions. Compared with the nitrification–denitrification pathway, the anammox pathway has the advantage of a lower demand for carbon sources [5,10,55]. The microorganisms involved in the anammox process have been identified to belong to the order *Brocadiales* of the phylum *Planctomycetes* [60,61]. However, no *Brocadiales* were detected by the analysis based on V3–V4 regions of the 16S rRNA gene in the current research. Nevertheless, the quantitative analysis of the anammox bacterial 16S rRNA gene, which is usually regarded as the marker of anammox process, clearly indicated the presence of anammox bacteria [55]. However, the absolute abundance of anammox bacteria was far lower than that of the *amoA* gene, which indicated that the ammonia process was not the dominant pathway of the ammonia nitrogen removal. This result was consistent with the opinion that nitrification–denitrification primarily contributes to the removal of ammonia nitrogen when the C/N ratio is less than or equal to 6:1 in the CWs [55].

Analysis of bacterial community composition revealed that Proteobacteria was the dominant phylum followed by Bacteroidetes in not only the plant rhizoplane, but also the rhizosphere in the current system. Proteobacteria has been widely considered an active participant in nitrogen removal in the CWs for its high diversity of metabolism which involves global carbon, nitrogen, and sulfur cycling [62]. The results of this study were consistent with several previous studies that indicated Proteobacteria as the dominant bacterial community in a variety of wetland substrates [48,49,62,63]. Further comparison of Proteobacteria by order also indicated a difference in the relative abundances of many orders, such as Burkholderiales, Oceanospirillales, Pseudomonadales, and Rhodocyclales, between samples from the substrate of planted systems and those from the unplanted ones. All the four mentioned orders have been reported to have a close relationship with denitrification, which indicates different characteristics of nitrogen removal between the planted and unplanted systems [41,64]. Moreover, a higher relative abundance of Bacteroidetes, which was presented by the genera *Flavobacterium* in the class *Flavobacteria*, in the planted systems may also relate to the nitrogen removal via the denitrification process [64]. In summary, the enhanced community diversity and evenness of the bacteria, especially denitrifying bacteria, in the planted systems, may play a significant role in the improved removal rate of TN from wastewater.

## 5. Conclusions

In conclusion, the presence of *O. javanica* had a significant facilitating effect on the nitrogen, especially TN, removal from wastewater in the FWS-CWS during the low-temperature season with mean water temperature lower than 10 °C. Because the macrophyte could provide extra organic carbon by root exudation and plant residues, it enhanced the microbial abundance, diversity, and evenness, as well as the abundances of *amoA*, *nosZ*, *nirS*, and *nirK*, and those closely related to nitrification–denitrification, on the rhizoplane and in the substrate. This suggests that FWS-CWS planted with *O. javanica* is a reliable option for a higher removal rate of nitrogen during low-temperature seasons. However, the increased COD concentration in planted systems may also cause secondary pollution and pose a new challenge for the wastewater purification, especially, when the wetland macrophytes begin to shrivel and die under low temperatures. Therefore, further studies on the selection of plant species, the control of plant density, as well as feasible improvements to the approach, for instance, adopting the FWS-CWS as part of an integrated wastewater treatment train, merit more attention in the future.

## Figures and Tables

**Table 1:** Characteristics of the influent at the beginning of 2nd, 4th, 6th, and 8th batch (means ± S.D., *p* < 0.05, *n* = 4).

Parameter	20 December 2015	19 January 2016	29 January 2016	18 February 2016
NH_4_^+^–N (mg·L^−1^)	8.9 ± 0.9	10.8 ± 0.4	10.8 ± 0.3	11.6 ± 0.5
NO_3_^−^–N (mg·L^−1^)	10.5 ± 0.8	10.8 ± 0.7	10..8 ± 0.9	10.4 ± 0.4
NO_2_^−^–N (mg·L^−1^)	0.650 ± 0.025	0.560 ± 0.025	0.380 ± 0.005	0.335 ± 0.005
TN (mg·L^−1^)	24.8 ± 1.5	25.7 ± 3.0	25.3 ± 0.9	26.6 ± 2.0
COD (mg·L^−1^)	14.4 ± 2.2	15.4 ± 2.5	14.6 ± 2.8	15.8 ± 1.8
DO (mg·L^−1^)	9.7 ± 0.8	8.9 ± 0.5	9.3 ± 0.9	8.3 ± 0.6
pH	7.88 ± 0.65	7.05 ± 0.45	6.45 ± 0.98	8.45 ± 1.24

NH_4_^+^–N: ammonia nitrogen; NO_3_^−^–N: nitrate nitrogen; NO_2_^−^–N: nitrite nitrogen; TN: total nitrogen; COD: chemical oxygen demand; DO: dissolved oxygen.

**Table 2:** Length, biomass, nitrogen content, root activity, and radial oxygen loss (ROL) rate in the plants (means ± SD., *p* < 0.05, *n* = 4).

Phase	System	Shoot Length (cm)	Root Length (cm)	Shoot Biomass (g·m^−2^)	Root Biomass (g·m^−2^)	N in Shoot (g·m^−2^)	N in Root (g·m^−2^)	Root Activity (μg TTC·g^−1^ Root·h^−1^)	ROL Rate (μmol O_2_·g^−1^ Root·h^−1^)
Initial	Tcw	50.0 ± 2.5	15.0 ± 1.0	95.0 ± 6.3	35.0 ± 3.6	1.425	0.315	48.5 ± 6.4	0.96 ± 0.045
	Tcp	50.0 ± 2.5	15.0 ± 1.0	95.0 ± 6.3	35.0 ± 3.6	1.425	0.315	48.5 ± 6.4	0.96 ± 0.045
2nd Batch	Tcw	52.0 ± 2.7	16.0 ± 1.4	86.0 ± 5.3	35.8 ± 5.5	1.290	0.322	42.6 ± 3.7	0.68 ± 0.097
	Tcp	52.0 ± 2.8	16.0 ± 1.3	84.0 ± 6.5	36.4 ± 3.7	1.260	0.328	46.7 ± 5.2	0.88 ± 0.098
4th Batch	Tcw	55.0 ± 3.5	17.0 ± 1.5	87.0 ± 5.8	37.0 ± 4.1	1.305	0.333	43.2 ± 5.3	0.79 ± 0.054
	Tcp	55.0 ± 3.4	17.7 ± 1.2	87.2 ± 7.6	38.3 ± 3.3	1.305	0.345	46.4 ± 5.5	0.87 ± 0.093
6th Batch	Tcw	54.0 ± 2.6	20.5 ± 2.0	92.3 ± 5.7	40.5 ± 5.2	1.385	0.365	42.8 ± 4.5	1.02 ± 0.065
	Tcp	53.0 ± 1.7	20.0 ± 2.4	91.6 ± 6.5	40.6 ± 5.1	1.374	0.365	47.5 ± 6.2	1.03 ± 0.061
8th Batch	Tcw	56.0 ± 2.5	22.6 ± 2.2	102.3 ± 13.5	46.8 ± 4.3	1.535	0.421	58.7 ± 6.5	1.28 ± 0.078
	Tcp	55.0 ± 2.5	23.0 ± 2.7	104.5 ± 14.4	48.0 ± 4.8	1.568	0.432	63.6 ± 8.9	1.48 ± 0.085

Tcw: FWS-CWS planted with *O. javanica* in substrate; Tcp: control systems without sands. The plant in each system was sampled at the initial time of the experiment and at the end of the 2nd, 4th, 6th, and 8th batches.

## Appendix A

**Table A1:** Primers of QPCR.

Target	Primer	Primer Sequence (5′-3′)	References
bacterial 16S rRNA gene	690F	TGTGTAGCGGTGAAATGCG	[65]
	829R	CATCGTTTACGGCGTGGAC	
archaeal 16S rRNA gene	ARC344F	ACGGGGYGCAGCAGGCGCGA	[66]
	ARC915R	GTGCTCCCCCGCCAATTCCT	
ANO 16S rRNA gene	AMX809F	GCCGTAAACGATGGGCACT	[67]
	AMX1066R	AACGTCTCACGACACGAGCTG	
*amoA*	*amo*1F	GGGGGTTTCTACTGGTGGT	[68]
	*amo*2R	CCCCTCKGSAAAGCCTTCTTC	
*nosZ*	*NosZ* 1527F	CGCTGTTCHTCGACAGYCA	[69]
	*NosZ* 1773R	ATRTCGATCARCTGBTCGTT	
*nirS*	*nirS* cd3AF	GTSAACGTSAAGGARACSGG	[70]
	*nirS* R3cd	GASTTCGGRTGSGTCTTGA	
*nirK*	*nirK* 583F	TCATGGTGCTGCCGCGKGACGG	[71]
	*nirK* 909R	GAACTTGCCGGTKGCCCAGAC	

**Table A2:** Detailed information for 16S rRNA gene qPCR and Illumina MiSeq sequencing analysis.

Information	qPCR	Illumina MiSeq Sequencing
Analysis system	Illumina-Eco real-time PCR system (Illumina, San Diego, CA, USA)	Illumina MiSeq 2500 sequencing platform (Illumina, San Diego, CA, USA)
Reaction mixture	5.0 μL SYBR^®^ Premix Ex Taq™ II (Takara, Otsu, Japan), 1.0 μL template DNA (diluted 100-fold), 0.5 μL forward and 0.5 μL reverse primers (10 μM), 3.0 μL RNase-free water	25 μL reaction mixture (including 10 ng template, 0.5 μL forward primer, 0.5 μL reverse primer)
PCR program	30 s at 94 °C, 40 cycles of 5 s at 95 °C, 30 s at 55 °C (*amoA*) or 60 °C (other genes), and 30 s at 72 °C	3 min at 94 °C, 30 cycles of 10 s at 94 °C, 15 s at 55 °C, and 72 °C for 30 s, and a final incubation at 72 °C for 7 min
PCR product purification	/	Agencourt AMPure beads (Beckman Coulter, Inc., Fullerton, CA, USA)
Libraries construction	/	NEBNext Ultra DNA Library Prep Kit for Illumina (New England Biolabs Inc., Boston, MA, USA)

**Table A3:** Alpha diversity analysis at 5% dissimilarity based on the 16S rRNA gene Miseq sequencing analysis.

Sample	OTUs	ACE	Simpson	Shannon-Even	Coverage
Ps	446	629.398681	0.219313	0.382270	0.994148
Pw	567	720.449124	0.137883	0.462411	0.994037
Sp	710	788.302182	0.031945	0.748674	0.995926
Su	675	761.131090	0.046425	0.695539	0.995444

The richness estimators (ACE), diversity indices (Simpson), evenness indices (Shannon-even), and coverage (Good’s coverage index) were calculated using the Mothur program. Ps: sample from rhizoplane in Tcw; Pw: sample from rhizoplane in Tcp; Sp: sample from sand in Tcw; Su: sample from sand in Tcs. The microbial samples from the plants rhizoplane and the substrate were obtained at the end of the 6th batch.

**Table A4:** The main genera (relative abundance >1.00%) with the addition of two nitrifying bacteria (*Nitrospira* and *unclassified-Nitrosomonadaceae*) in the samples from rhizoplane or sand.

Phylum	Genus	Read Numbers				Relative Abundances (%)			
		Ps	Pw	Sp	Su	Ps	Pw	Sp	Su
Bacteroidetes	*Anaerorhabdus*	0	5	278	98	0.00	0.02	1.03	0.36
Bacteroidetes	*Chryseobacterium*	224	68	5	8	0.83	0.25	0.02	0.03
Bacteroidetes	*Flavobacterium*	76	181	752	321	0.28	0.67	2.79	1.19
Bacteroidetes	*Lutibacter*	0	5	293	366	0.00	0.02	1.09	1.36
Bacteroidetes	*Sunxiuqinia*	2	3	274	28	0.01	0.01	1.01	0.10
Bacteroidetes	*unclassified-Bacteroidetes*	13	31	1129	509	0.05	0.11	4.18	1.89
Cyanobacteria/Chloroplast	*Bacillariophyta*	4	43	328	33	0.01	0.16	1.21	0.12
Firmicutes	*Bacillus*	465	52	73	79	1.72	0.19	0.27	0.29
Firmicutes	*Exiguobacterium*	2401	1387	8	4	8.89	5.14	0.03	0.01
Firmicutes	*Paenibacillus*	21	792	4	1	0.08	2.93	0.01	0.00
Firmicutes	*Trichococcus*	5	0	1	0	0.02	0.00	0.00	0.00
Ignavibacteriae	*Ignavibacterium*	2	1	261	302	0.01	0.00	0.97	1.12
Planctomycetes	*Planctomyces*	4	13	3	3	0.01	0.05	0.01	0.01
Proteobacteria	*Acidovorax*	81	255	9	30	0.30	0.94	0.03	0.11
Proteobacteria	*Aeromonas*	658	1952	55	21	2.44	7.23	0.20	0.08
Proteobacteria	*Arcobacter*	5	1	0	1	0.02	0.00	0.00	0.00
Proteobacteria	*Arenimonas*	24	56	1350	1353	0.09	0.21	5.00	5.01
Proteobacteria	*Azoarcus*	27	7	311	339	0.10	0.03	1.15	1.26
Proteobacteria	*Azospira*	0	6	42	44	0.00	0.02	0.16	0.16
Proteobacteria	*Azospirillum*	12	4	41	9	0.04	0.01	0.15	0.03
Proteobacteria	*Bradyrhizobium*	10	39	23	20	0.04	0.14	0.09	0.07
Proteobacteria	*Dechloromonas*	37	69	85	61	0.14	0.26	0.31	0.23
Proteobacteria	*Geobacter*	0	1	335	106	0.00	0.00	1.24	0.39
Proteobacteria	*Halomonas*	74	129	3911	5044	0.27	0.48	14.49	18.68
Proteobacteria	*Hydrogenophaga*	31	44	492	752	0.11	0.16	1.82	2.79
Proteobacteria	*Hyphomicrobium*	2	6	23	41	0.01	0.02	0.09	0.15
Proteobacteria	*Limnobacter*	1	2	44	174	0.00	0.01	0.16	0.64
Proteobacteria	*Mesorhizobium*	4	11	41	20	0.01	0.04	0.15	0.07
Proteobacteria	*Paraperlucidibaca*	0	1	252	19	0.00	0.00	0.93	0.07
Proteobacteria	*Perlucidibaca*	10	7	664	790	0.04	0.03	2.46	2.93
Proteobacteria	*Porphyrobacter*	59	92	207	80	0.22	0.34	0.77	0.30
Proteobacteria	*Pseudomonas*	20,068	17,756	1402	2603	74.33	65.76	5.19	9.64
Proteobacteria	*Pseudoxanthomonas*	1	2	6	6	0.00	0.01	0.02	0.02
Proteobacteria	*Rhizobium*	163	272	50	48	0.60	1.01	0.19	0.18
Proteobacteria	*Rhodobacter*	44	73	253	176	0.16	0.27	0.94	0.65
Proteobacteria	*Rhodoferax*	39	78	2088	863	0.14	0.29	7.73	3.20
Proteobacteria	*Sandaracinobacter*	0	0	84	307	0.00	0.00	0.31	1.14
Proteobacteria	*Shewanella*	0	17	5	1	0.00	0.06	0.02	0.00
Proteobacteria	*Simplicispira*	13	19	522	393	0.05	0.07	1.93	1.46
Proteobacteria	*Stenotrophomonas*	0	4	1	2	0.00	0.01	0.00	0.01
Proteobacteria	*Sulfuritalea*	1	5	207	644	0.00	0.02	0.77	2.39
Proteobacteria	*Thauera*	1	4	94	179	0.00	0.01	0.35	0.66
Proteobacteria	*Thiobacillus*	1	0	367	463	0.00	0.00	1.36	1.71
Proteobacteria	*unclassified-Rhodobacteraceae*	66	165	594	496	0.24	0.61	2.20	1.84
Proteobacteria	*unclassified-Sphingomonadales*	27	43	202	352	0.10	0.16	0.75	1.30
Proteobacteria	*unclassified-Xanthomonadaceae*	8	15	219	855	0.03	0.06	0.81	3.17
Nitrospirae	*Nitrospira*	0	1	3	2	0.0000	0.0037	0.0111	0.0074
Proteobacteria	*unclassified-Nitrosomonadaceae*	1	2	24	9	0.0037	0.0074	0.0889	0.0333

Possible denitrifying bacteria according to Heylen et al. (2006) and Philippot et al. (2007) in each sample are given in bold. Ps: sample from rhizoplane in Tcw; Pw: sample from rhizoplane in Tcp; Sp: sample from sand in Tcw; Su: sample from sand in Tcs.
